# Dental Implant Placement in Medically Compromised Patients: A Literature Review

**DOI:** 10.7759/cureus.54199

**Published:** 2024-02-14

**Authors:** Walla Samara, Omid Moztarzadeh, Lukas Hauer, Vaclav Babuska

**Affiliations:** 1 Department of Stomatology, University Hospital Pilsen, Faculty of Medicine in Pilsen, Charles University, Czech Republic, Pilsen, CZE; 2 Department of Anatomy, Faculty of Medicine in Pilsen, Charles University, Czech Republic, Pilsen, CZE; 3 Department of Medical Chemistry and Biochemistry, Faculty of Medicine in Pilsen, Charles University, Czech Republic, Pilsen, CZE

**Keywords:** osseointegration, medically compromised patients, dental implant failure rate, dental implants, systemic disorders

## Abstract

As a discipline of dentistry, oral implantology deals with the diagnosis, design, insertion, restoration, and/or management of alloplastic or autogenous oral structures for the purpose of regaining contour, function, aesthetics, and speech in a partially or completely edentulous patient. The present review aims to provide the currently available knowledge about the impact of certain systemic disorders and the usage of some medications on the survival rate of dental implant therapy and to highlight the importance of patient management under these conditions. Diabetes, osteoporosis, cardiovascular diseases, and the intake of some medications can increase the risk of the failure of a dental implant. Even though there are relatively few medical contraindications to dental implant treatment, certain conditions may increase the risk of failure or complications.

## Introduction and background

Nowadays, dental implants are a very common procedure, as they are highly dynamic and functional restorations for replacing missing teeth. Though many studies discussed the success and high survival rate of dental implants, failures continue to happen for many causes. Failure of dental implants can be attributed to the lack of bone healing response following dental implant surgery in some medical disorders [1,2]. It is noteworthy that although early failure is common, the success of implantation following prosthetic loading has largely been discussed, which restricts our understanding of the causes of preloading failures. Implant location, dimensions, bone volume and quality, systemic diseases, and immune factors contribute to early implant failure among other variables [3]. Improper position of implant placement or osseointegration failure may activate the host's inflammatory response and result in peri-implantitis, which has become a rising issue in the dental field due to an absence of effective treatment options [4]. Several risk factors have been associated with periodontal and peri-implant diseases. The risk factors for periodontal disease are uncontrolled diabetes, obesity, osteoporosis, and malnutrition while for peri-implantitis, the risk factors include a previous history of periodontitis, and poor plaque control [5]. Biological problems can emerge and limit the lifespan of dental implants. Peri-implantitis has been identified as the leading cause of implant failure, it is an inflammatory disorder characterized by tissue degradation, and if not treated effectively, it is likely to develop into bone loss [6]. Recent clinical studies have shown that smoking, bad oral hygiene, and postoperative infections can contribute to the failure of osseointegration [7]. Chrcanovic et al. assessed the influence of some systemic conditions, such as hypertension, cardiac diseases, and diabetes mellitus, on early implant failure, and results showed that failure rates were not that significant and no definitive conclusion was obtained [8]. In a retrospective study by Javed and Romanos, results revealed that patients with systemic diseases could have good survival rates, similar to healthy individuals [9]. Some systemic disorders are classified as absolute contraindications for implant treatment such as recent myocardial infarction or cerebrovascular accident, high risk of bleeding, significant immunosuppression, active cancer therapy, and intravenous bisphosphonate treatment [10]. Other systemic disorders are classified as relative contraindications, including diabetes mellitus, osteoporosis, hypothyroidism, and smoking [11]. According to the British Dental Journal, implant failures are divided into four groups: failure due to loss of integration, positional failures, soft tissue failures, and biomechanical failures [12]. Nevertheless, the survival of dental implant therapy in medically compromised patients still can’t be expected [13]. Therefore, the present review aims to provide recently available knowledge about the impact of certain systemic disorders and the most commonly used medications on dental implant therapy and to highlight the importance of patient management under these conditions.

## Review

Systemic disorders

Diabetes Mellitus

Diabetes mellitus is a chronic metabolic disorder characterized by a high blood glucose level, either because of impaired insulin secretion or insulin resistance by the cells [14]. Type 1 diabetes is due to a deficiency in insulin secretion because of destroyed pancreatic-producing cells. Type 2 diabetes occurs in most cases due to insulin resistance, but impaired insulin secretion may appear with time. Prediabetes is a condition where there is an abnormal blood glucose level, where it is higher than normal but not to the level that can be diagnosed as diabetes [9]. Diagnostic tests for diabetes according to American Dental Association are summarized in Table 1.

**Table 1:** Diagnostic criteria for diabetes according to the American Diabetes Association

Diagnostic test	Normal	Prediabetes	Diabetes Mellitus
A1C (glycated hemoglobin)	<5.7%	5.7 - 6.4 %	≥6.5% (48 mmol/mol)
2-h PG (plasma glucose)	<140 mg/dL	140 - 200 mg/dL	≥200mg/dL (11.1 mmol/L)
FPG (fasting plasma glucose)	<100 mg¤dL	100 - 125 mg¤dL	≥126 mg/dL (7.0 mmol/L)
Random plasma glucose in a patient with classic symptoms of hyperglycemia or hyperglycemic crisis	N/A	N/A	≥200 mg/dL (11.1 mmol/L).

**Figure 1:**
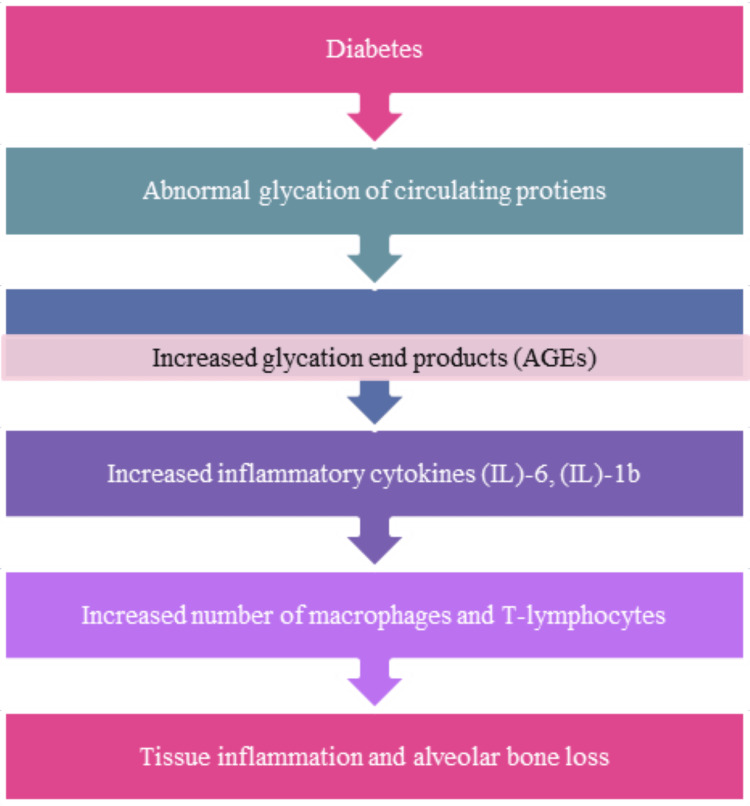
A possible mechanism of diabetes-related alveolar bone loss in dental implant therapy The figure is the author's own creation.

Under stable conditions, when an implant is installed, a blood clot forms and vascularizes, mesenchymal stem cells (MSCs) multiply and differentiate into osteoblasts, and bone remodeling occurs. Diabetes may alter bone cell metabolism, interfering with bone healing and reducing bone matrix strength, leading to reduced bone density and decreased bone quality, thus increasing the possibility of osteoporosis and fractures. Given these findings, it is reasonable to tell that diabetes mellitus might negatively affect osseointegration [15-16]. High glucose levels in the bloodstream can cause abnormal glycation of circulating proteins and increase the production of advanced glycation end products (AGEs). There is a belief that the medical syndrome causes plasma free fatty acids (FFAs) to go up. High blood levels of FFAs may also impair glucose metabolism due to the predominant mitochondrial oxidation of lipids, causing glucose uptake to drop and creating chronic hyperglycemia. As glucose levels rise, circulating proteins can get pathologically glycated, and advanced glycation end products (AGEs) can form and these will produce inflammatory cytokines such as interleukin (IL)-6, (IL)-1b, as well as an increase in inflammatory macrophages and T-lymphocytes, resulting in tissue inflammation and bone loss (Figure 1) [17,18].

Chronic hyperglycemia leads to micro and macroangiopathy. Patients have impaired wound healing, a high recurrence rate of periodontitis, recurrent tooth loss, and a high risk of infections. A long time ago, it was believed that diabetic patients had some restrictions to receiving implant therapy. However, recent studies have proved the advantage of implant therapy for diabetic patients [19]. Modified implants with acid etch and sandblasting treatment appear to have the ability to increase osseointegration and have an increased potential for bone formation in type 2 diabetes mellitus (T2DM) rats when compared with the traditional machined surface [20]. Clinical peri-implant parameters, bleeding on probing (BOP), and radiographic bone loss were found to be at a higher level in diabetic and pre-diabetic patients than non-diabetic, and probing depth (PD) was extensively higher in diabetic patients, thus leading to less osteointegration of the dental implant [21]. A clinical study by Granato et al. assessed the significance of using a modified dental implant surface treated with bioactive calcium phosphate (CaP) on osseointegration in patients with metabolic syndrome conditions, especially with T2DM, as compared to healthy individuals. Results have shown that there is no great difference in the mechanical parameters. While the histomorphic parameters of bone are lower for patients with metabolic syndrome, they have a lower amount of bone around the implant [18]. Several cofounder factors should be carefully considered when assessing dental implant success, including quality of bone, class of occlusion, and angulations of implants. Further longitudinal studies are needed to confirm the results regarding this issue [22]. Furthermore, the preservation of a healthy gingiva through regular non-invasive debridement of plaque could help in decreasing inflammation in diabetic patients [17]. Antibiotics and chlorhexidine are commonly prescribed as perioperative anti-infective therapy nowadays to ensure implant success. It is still unknown whether dental implants in patients with prediabetic conditions have a negative impact on the development of peri-implant diseases, though they still have no impact on the survival of implants [23]. Based on some recent studies, results have shown that patients with well-controlled diabetes mellitus could have a success rate of dental implants similar to healthy individuals. Until this moment, studies didn’t provide sufficient proof of the success of dental implants in diabetic patients. However, it is hypothesized that the usage of immediate dental implant techniques could be helpful for these patients as long as they have controlled T2DM [2,24].

Osteoporosis

Osteoporosis is a metabolic bone disorder associated with an excessive decrease in bone density and impaired bone structure [16]. It commonly occurs in old age people, but it’s also identified as a relative contraindication for dental implant therapy due to impaired quality of bone and alveolar ridge atrophy caused by osteoporosis, as the main negative side effect is the high risk of fractures [25,26]. It’s been strongly advised that antibiotics should be considered before the operation for patients who are taking anti-resorptive drugs, thus, more healing interval time should be given before inserting the implant prosthesis inside [27]. There is evidence that the use of bisphosphonates (BP), especially parenteral bisphosphonates, which are the most frequently prescribed pharmacologic agents for the treatment of osteoporosis, may accelerate the rate of failure of the osseointegration of implants [3].

Current literature highlighted the importance of utilizing modified types of dental implant surfaces since it’s been found that treated surfaces may help in lowering bone tension and increasing osseointegration in these patients as compared to machined surfaces. More clinical studies should be done to confirm these results [28]. For osteoporotic patients who are taking fewer doses of anti-resorptive drugs, a dental implant is the choice of treatment, as the risk of osteonecrosis is lower, unlike other patients with severe malignancies where dental implants are not allowed due to their taking high doses of these drugs [29]. Osteoporosis is very common to occur in postmenopausal women, but recent studies have shown high survival rates of implant therapy in osteoporotic women, between 93.8% and 100%. Given these results, they suggested that osteoporosis doesn’t contradict dental implant therapy [30]. Studies reported that the healing process of fractures that occur due to osteoporosis is still sufficient despite the bone changes caused by the disease [10]. Mardas et al. reported in their study that usage of modified sandblasted/acid-etched (SLA) surface titanium may have a positive influence on bone healing and bone-to-implant contact of tested osteoporotic animals [31]. Antiresorptive drugs like bisphosphonates work mainly on slowing down the action of osteoclast cells, lowering the bone remodeling process, and leading to osteonecrosis of the jaw (ONJ) in the long term, which results in the failure of implant osseointegration. Therefore, this remains a matter of debate in the literature [32]. In previous clinical studies, a success rate (97%) of dental implants was shown in osteoporotic patients after a long time of follow-up [33]. A recent systematic review has summarized all studies that have been conducted on implant placement in osteoporosis patients, and results revealed that regional bone density is a useful parameter as a predictor of primary implant stability. Careful evaluation of bone mineral density can improve the prognosis for dental implants in patients with osteoporosis [34]. According to most of the studies that were reviewed by Sbricoli et al., the results showed no difference between patients with metabolic bone disease and healthy patients in the survival rates of dental implants, so there isn't enough evidence to argue that osteoporosis increases peri-implantitis risks [35]. A study by Shibili et al. compared the histological properties of implants in patients with and without osteoporosis; neither group had different percentages of bone-implant contact and results weren't different between groups either after osseointegration was achieved. According to these results, osteoporosis doesn't seem to be a contraindication for implant placement in osteoporotic patients [36]. Given these reports, it’s concluded that dental implant therapy could be an option for osteoporotic patients.

Cardiovascular Diseases (CVDs)

Medical literature identified cardiac diseases as a relative contraindication for dental implant therapy, as there is a high risk of developing infective endocarditis. Patients with cardiovascular disorders are expected to have diminished blood flow, and thus, this leads to a lower oxygen level and fewer amounts of nutrients in bone tissues. Therefore, it is thought that the failure of osseointegration will be much higher [30,37]. Patients with CVDs are vulnerable to a high risk of infections due to chronic hypoxia, which disturbs the immune response and decreases the action of macrophage cells. Precise observation and systematic control of medication intake are highly required in these patients. A prophylactic antibiotic prescription may be needed for infective endocarditis prevention [16]. Occurrence of bleeding or sudden cardiac ischemia at the time of surgery should be considered and consultation should be taken before starting implant surgery [38]. The American Heart Association suggests antibiotic prophylaxis for patients who have prosthetic heart valves, heart transplants, and congenital heart diseases. Antibiotics should be administered 30-60 minutes before invasive dental procedures that may include surgeries and piercing oral soft tissues [39]. Drug therapy can be a critical cause of uncontrolled bleeding; thus, bleeding time and international normalized ratio (INR) should be carefully monitored for patients receiving oral anticoagulants (aspirin, warfarin, clopidogrel) for cardiovascular illness. The normal INR in these kinds of patients should be 2.2 or less for surgical procedures and the normal platelet count is between 100,000 and 500,000/m^3^ [10]. Kaura et al. found in their clinical study that it's safe to place dental implants in patients taking single anti-platelet drugs like aspirin and clopidogrel without stopping them but when a couple of anti-platelets are used, the bleeding risk would be mild to moderate. Thus patients on dual anti-platelet drugs must consult their cardiologist before having dental surgery [40]. In a review paper by Aghaloo et al. and co-workers, failure rates of dental implants were observed in a different group of patients, and they found that there was no big difference in rates of failure between healthy groups and other patients with cardiovascular disorders [32]. In a 2016 systematic review, only two out of five studies found that cardiovascular diseases affect implant osseointegration and lead to inflammation of the bone, though the number of patients was small [41]. On the contrary, the results of a retrospective study by Neves and his colleagues showed that patients who have cardiovascular diseases developed higher rates of implant failure among 721 medically compromised patients who were included in the study [42]. A recent study reported that the extent of peri-implantitis was not that critical (6.8%) in patients with cardiovascular disease as compared to patients without a background of cardiovascular diseases [43]. A 2020 clinical study by Ustaoğlu et al. aimed to investigate serum biochemical parameters, also known as cardiovascular risk markers, in people with dental implants, and to find out if peri-implant diseases are prevalent. Clinical parameters surrounding the implant were positively correlated with triglyceride and uric acid levels, as the peri-implantitis group had significantly higher levels of triglycerides and uric acid. It's important to check patients' serum biochemical parameters before implant surgery [44]. Yet, the evidence regarding the effect of cardiovascular disease and its biological complications on dental implants is still debatable and inconclusive [45].

Hypertension

Hypertension is a chronic medical condition in which blood pressure in the arteries is elevated. In the case of dental implant patients who use antihypertensive medications, assessing how these drugs affect osseointegrated implants is crucial. In a retrospective study, where 35 patients received 77 anodized dental implants and the impact of antihypertensive medications on peri-implant tissue parameters was evaluated, the results showed notable variation in the probing pocket depth and having peri-implantitis between users and non-users, thus indicating an association between the usage of antihypertensive drugs and clinical characteristics in anodized peri-implant tissue [46].

On the other hand, in a systematic review where 70 publications were selected for the review of the role of many prevalent systemic illnesses, including hypertension and cardiovascular disease, in the development of peri-implantitis. Most of the research findings indicate that there is no correlation between the risk of peri-implantitis with cardiovascular disease and hypertension [35]. A few bibliographic studies have been published on hypertension and peri-implantitis, like Wu et al.'s in 2016. They studied 325 implants and only found 0.6% of complications. As it’s known antihypertensive drugs are good for bone formation and bone remodeling, and they help keep bone fractures to a minimum, antihypertensive drugs may make implants more likely to survive [43]. A retrospective study examined the effects of chronic intake of some medications, including antihypertensive drugs, on dental implant survival and peri-implantitis. Results didn't show any correlation with antihypertensive drugs. Though such a drug category is widely used worldwide, there's very little literature on it. Wu et al. found that antihypertensive medicines improved implant survival rates in one study involving 1499 dental implants in 728 patients [47,48].

Thyroid Disorders

Thyroid disorders are the second most common endocrine disease mainly affecting females and appear in 1% of the population. Thyroxine hormones are T3 and T4, which are produced by the thyroid gland and control the metabolism of fats, carbohydrates, and proteins. Thyroxine is also necessary for healthy bone turnover and enhances the effects of other hormones, including growth hormone and catecholamines [49]. Hyperthyroidism is associated with increased T3 and T4, mainly caused by Graves’ disease and toxic adenoma. It has a detrimental effect on bone mass due to a high bone turnover, as documented by a shortened bone remodeling cycle, together with an increase in biochemical markers of bone resorption and bone formation, producing an increase in mineral apposition and formation, as well as a decrease in bone mineral density [50]. Hypothyroidism, a condition when the thyroid gland is unable to synthesize enough hormones to meet the needs of the entire body, is another important metabolic problem. Thyroid hormone stimulates the synthesis of insulin-like growth factor 1, which promotes osteoblast development and maintains adult bone mass while also influencing bone metabolism [51]. A 2022 systematic review showed that patients with thyroid disorders can get dental implants with similar survival rates as patients without thyroid disorders, so they can be rehabilitated with dental implants. Most of the articles evaluated the implant survival rate with a mean of 92.56% for patients with thyroid disorders. Hence, more studies and bigger sample sizes are required. The lowest recorded dental implant survival percentage (71.2%) was found in a study by De Souza JG et al. who linked this prevalence to a history of periodontitis rather than the thyroid condition [50]. A review by D’Ambrosio et al. aimed to find out drugs and diseases that can affect bone-implant integration. Eight systematic reviews were included in the review, and the results showed that hypothyroidism didn't lead to a lower implant osseointegration rate. According to Aghaloo et al.'s study, hypothyroidism has been linked to a higher risk of fracture as well as a delayed rate of bone regeneration; however, results didn’t show statistically significant variations in the rate of implant failures in hypothyroid patients compared to healthy subjects [51].

Medications

Bisphosphonates

Bisphosphonates (BP) are a class of medications that mainly inhibit bone resorption, used in the treatment of bone diseases such as osteoporosis and Paget’s disease [52]. Bisphosphonate’s work is based on increasing osteoblast rate and promoting osteoclast apoptosis. In Migliorati et al.'s 2003 study, results showed that patients who received IV bisphosphonates had the potential to develop osteonecrosis of the jaw. Because of this risk, these patients are not recommended to go through surgical procedures including dental implant therapy [7,53] Though the pathophysiology of bisphosphonate-related osteonecrosis of the jaws (BRONJ) is not fully known, it’s suggested that when osteoclast cells are blocked, the bone remodeling process will be damaged, which is crucial for bone healing, thus leading to more complications of bone exposure [54]. It has been suggested that a history of oral bisphosphonate use for a short time could help in reducing the risk of bone necrosis. Further studies are required to confirm the results [55]. Thus, it's recommended for patients who are using oral bisphosphonates to take a prophylactic antibiotic before any invasive procedure, such as dental implant placement, like penicillin or metronidazole, combined with a quinolone in case of a penicillin allergy [56]. On the other side, in a 2008 systematic review by Grant et al., results showed that implant therapy outcomes in patients with oral bisphosphonates use were similar to those without oral bisphosphonates use and there was no mention of developed bone necrosis after implant treatment [57]. Furthermore, an interesting result by Chappuis et al. found that oral BPs have no important role in dental implant failure, though most studies suggest the opposite [58]. As a result of a review with a total of 11 articles, the activity of bisphosphonates-releasing titanium implants has been examined, indicating that nine studies (82%) have shown greater osseointegration and bone formation, whereas only two studies (18%) have reported that there is no benefit to using BP-coated implants, so more clinical studies are needed to confirm these results [59].

The influence of bisphosphonates on bone-implant contact in a rabbit model treated by intravenous BPs has been analyzed. Results have shown that neither the density nor microstructure of bone is affected and it has a negative impact on the osseointegration process in the near future as it starts to be less with time [60]. Furthermore, in a retrospective cohort study by French et al. (2019), they reported that bisphosphonate therapy does not affect dental implant success. Despite this, there was an important risk of bone loss over time. This is an unusual finding in the literature and could reflect altered bone remodeling potentials, where sudden bone loss was observed in some but not all bisphosphonate-related patients [61]. In a 2020 clinical study, 80 patients who are anti-resorptive drug users with 344 dental implants who had more than 12 months of follow-up from the beginning of drug treatment were included, along with 80 non-anti-resorptive drug users from the same interval of time. Results have shown that the success rate of implants was 89.83% among patients taking anti-resorptive drugs and 96.03% among non-users and the possibility of implant failure decreased when the interval between implant placement and drug intake was less than 36 months [62]. In a recent systematic review including different studies for a total of 378 patients who had dental implants, the outcome of dental implant therapy in some cases was positively improved when bisphosphonates were consumed; however, further clinical studies are required, as well as longer follow-ups, to fully clarify this doubt [63].

Immunosuppressive and Nonsteroidal Anti-Inflammatory Drugs (NSAIDs)

Having a good immune response is essential for wound healing after each surgery. One of the most common immunosuppressive drugs is Cyclosporine A (CsA). It has been thought that CsA could affect the bone remodeling process and decrease the action of osteoblast cells, thus affecting the possibility of bone healing [7,10]. In a recent prospective cohort study, results have shown high survival rates (98%) of implant therapy in the long run in immunosuppressed patients after renal transplantation [13]. In a current clinical study with three months of follow-up, the authors reported that patients who are on immunosuppressive drugs have good bone healing around dental implants, and there is no mind of having implants as an option of treatment, though there are not enough reported results about the success of implants for these patients [41]. A successful clinical case of dental implant placed for a patient with myelogenous leukemia treated by an improved kind of anti-cancer immunosuppressive drug (nilotinib) has been reported by Morita et al. It’s thought that these molecular-targeted medications reduce the incidence of immunosuppression, which could not be done with classical drugs, thus negative signs like the movement of the implant and peri-implantitis have not been seen in this clinical case [64]. Moreover, the success of dental implants with no problems has been mentioned for patients with autoimmune diseases like pemphigus vulgaris, as they are treated by corticosteroids, although it’s believed that these drugs, in the long run, can lower immunity and affect bone healing. However, it’s important to highlight that these patients should be covered by additional corticosteroids before operations like oral surgery due to their effect on the adrenal gland [65]. Some studies had been done on animal models infused by shots of Cyclosporins, and on X-ray, they summarized that bone remineralization and healing are negatively affected [66]. Recent studies have shown that the intake of CsA before implant surgery will affect the osseointegration around dental implants and lead to impaired quality of bone, resulting in a decrease in the success rate of implant therapy [67]. In the Durate et al. study (2001), results showed that the use of CsA increases bone loss and impairs bone healing around dental implants [68]. Furthermore, some findings showed that patients who receive CsA after transplant surgery have the potential to develop osteoporosis [69]. Based on recent findings about the influence of immunosuppressive drugs on bone, they should be carefully considered when it is related to the survival of implants [7]. NSAIDs are one of the most used medications. NSAIDs such as cyclooxygenase-2 (COX-2) and selective inhibitors (selective NSAIDs) have antipyretic, analgesic, and anti-inflammatory effects, while aspirin has remarkable antiplatelet aggregating activity [70]. Some NSAIDs, including ibuprofen and flurbiprofen, are commonly used for relieving pain after implant surgery due to their anti-inflammatory and analgesic effects [7]. These kinds of drugs act mainly on the inhibition of the cyclooxygenase (COX) enzyme, thus leading to the suppression of prostaglandin formation, which has an important role in bone construction and normal bone healing [71]. Most of the recent literature believes that the level of NSAIDs’ negative impact on bone healing and osseointegration relies on how much they can inhibit cyclooxygenase enzyme activity, as this only can be done by some types of NSAIDs [72]. A 2016 retrospective study by Winnet et al. showed that 50% of patients treated with NSAIDs had the lowest success rates of dental implants, though it is worth saying that differences in types and number of drugs could affect these results. Furthermore, results showed that patients who received NSAIDs before implant surgery had more failures and increased bone loss around implants [73].

In a recent 2017 study, they tried to assess how NSADIs influence bone-implant contact in the use of a modified implant surface. Results showed that the effects of these drugs relied on many factors, including the dose of the drug and there was no significant effect on bone growth at least for the drugs used in the study. Furthermore, it was noticed that hydroxyapatite (HA)-coated implant surfaces had good bone-implant contact [74]. Based on most long-term studies that were done on a large number of implant cases, results showed that patients who are NSAID users had higher rates of failures and bone loss than those who are not [57]. Interestingly, a study conducted on rabbit models was conducted to evaluate the effect of diclofenac sodium and parecoxib on bone-implant contact in the calvarial bone. In contrast to other studies, these results showed that these drugs did not have any significant effects on bone [75]. Yet, more studies are required in the presence of a smaller number of human studies on this topic [74].

Antidepressants

Depression is a mental illness that reduces quality of life and causes significant disabilities. Depression has been linked to low levels of serotonin, and selective serotonin reuptake inhibitors (SSRIs) have been used to treat it in recent years [7]. It's been shown in some clinical studies that these kinds of medications lead to lower bone mineral density and more bone fractures [51]. Serotonin receptors are present in peripheral tissues like the digestive tract, blood platelets, as well as osteoblasts and osteoclasts. As a result of blocked serotonin reuptake, osteoclast differentiation increases, and osteoblast proliferation is less, and this leads to decreased bone density. A retrospective study was conducted by Altay et al., including a total of 2055 osseointegrated dental implants in 631 patients. Researchers found that SSRIs might cause osseointegration failure but are not statistically significant [76].

A comprehensive systematic review by D’Ambrosio et al. showed that there were more dental implant failures in people taking SSRIs. Based on Chappuis et al.'s review, SSRI users have a 7.5% higher failure rate than non-SSRI users. Similarly, Aghaloo et al.'s review revealed that dental implant survival rates ranged from 89.4% to 94.4% among people with SSRIs compared to 95.4% to 98.15 % among non-users of SSRIs. [51]. In a meta-analysis, implant survival was studied in patients with neuropsychiatric and neurodegenerative disorders, and seven studies were included in the systematic review. Analysis showed that patients without any disorders or taking medications for these disorders were significantly more likely to survive the implants and showed that patients who didn't take SSRIs had better implant survival than those who did. Also, it’s worth mentioning that any type of neuropsychiatric or neuromuscular disorder can limit proper oral hygiene and eventually contribute to early implant failure [77].

A recent retrospective study by Pena et al. has shown that the consumption of SSRI has been associated with an increased risk of dental implant failure, and these drugs are associated with a 4.53 times higher rate of dental implant failure. It’s worth considering that the increased risk of dental implant failure might be more related to the patient's psychological condition than to SSRI intake. Depressive illnesses can adversely affect oral health, resulting in a lack of dental care and lower cooperation. As a result, peri-implant diseases are more common [78].

## Conclusions

In the present study, the influence of different risk factors, including osteoporosis, diabetes, cardiovascular diseases, and thyroid disorders, on dental implant failure has been reviewed. Cardiovascular conditions and uncontrolled diabetes are considered higher risks for dental implant failure due to their adverse effects on osseointegration. Osseointegration can also be complicated by some medications, including bisphosphonates, NSAIDs, antidepressants, and immunosuppressants. Coated dental implants offer a great advantage and reduce the possibility of dental implant failure. Risks related to dental implant therapy can be a significant difficulty to the patient, hence further studies are required to confirm the mentioned results regarding this topic.
